# Multiplex methods provide effective integration of multi-omic data in genome-scale models

**DOI:** 10.1186/s12859-016-0912-1

**Published:** 2016-03-02

**Authors:** Claudio Angione, Max Conway, Pietro Lió

**Affiliations:** School of Computing - Teesside University, Middlesbrough, UK; Computer Laboratory - University of Cambridge, Cambridge, UK

**Keywords:** Genome-scale models, Multiplex networks, Multi-omics, Network fusion, Transcriptomics, Fluxomics, Flux balance analysis

## Abstract

**Background:**

Genomic, transcriptomic, and metabolic variations shape the complex adaptation landscape of bacteria to varying environmental conditions. Elucidating the genotype-phenotype relation paves the way for the prediction of such effects, but methods for characterizing the relationship between multiple environmental factors are still lacking. Here, we tackle the problem of extracting network-level information from collections of environmental conditions, by integrating the multiple omic levels at which the bacterial response is measured.

**Results:**

To this end, we model a large compendium of growth conditions as a multiplex network consisting of transcriptomic and fluxomic layers, and we propose a multi-omic network approach to infer similarity of growth conditions by integrating layers of the multiplex network. Each node of the network represents a single condition, while edges are similarities between conditions, as measured by phenotypic and transcriptomic properties on different layers of the network. We then fuse these layers into one network, therefore capturing a global network of conditions and the associated similarities across two omic levels. We apply this multi-omic fusion to an updated genome-scale reconstruction of *Escherichia coli* that includes underground metabolism and new gene-protein-reaction associations.

**Conclusions:**

Our method can be readily used to evaluate and cross-compare different collections of conditions among different species. Acquiring multi-omic information on the topology of the space of experimental conditions makes it possible to infer the position and to build condition-specific models of untested or incomplete profiles for which experimental data is not available. Our weighted network fusion method for genome-scale models is freely available at https://github.com/maxconway/SNFtool.

**Electronic supplementary material:**

The online version of this article (doi:10.1186/s12859-016-0912-1) contains supplementary material, which is available to authorized users.

## Background

As the cost of collecting omic data is likely to decrease in the coming years, methods to integrate different types of biological data are likely to become increasingly important. Biological data is often expressed as network data. As a result, methods to tackle networks with different layers, each representing a different omic, are likely to play a key role to integrate these data types. A common approach in this case is a layer-wide analysis of each layer separately, finally integrating the separate results into a single aggregate measure.

However, integrating results collected on single layers separately may cause loss of information, especially in cases where the inter-layer interactions are non-negligible. Intuitively, by analyzing different types of data in isolation we miss the information that is implicit in the coordinated activity of the different layers. For instance, in an application to networks of patients to investigate cancer, this approach resulted in different clusters of patients depending on which dataset was used, therefore causing incorrect subdivision of patients or samples in different molecular cancer subtypes [1].

Genome-scale models of biological organisms are often used to analyze the metabolic potential and to identify the metabolic interventions required to produce metabolites of interest. In this post-genomic era, more than 90 genome-scale metabolic reconstructions have been published to date [2], for both prokaryotes and eukaryotes. Computational methods on genome-scale models make it possible to explore the reaction network and find solutions that take into account noncomparable objectives, while satisfying all the given constraints.

With the growing availability of multi-omic databases, model-building and data integration techniques, a number of methods for integration of omic data have been proposed recently (for a comprehensive review and current challenges, the reader is referred to Saha et al. [3] and Bordbar et al. [4]). The wide variation in response of a single organism across differing conditions is facilitated by complex metabolic networks, which often include multiple levels of redundancy and adaptability. This is evident in the heterogeneity at the transcriptomic, proteomic and fluxomic levels across conditions [5]. In this regard, evaluating and aggregating in a single network the response to growth condition on different omic layers, while accounting for the dependence between the metabolic response at different levels (e.g. transcriptomic and fluxomic), is a highly desired feature, as it increases the reliability of any comparative analysis performed on the conditions, and provides overall topological information.

Our idea is to integrate multiple data types, collected from a comprehensive set of environmental conditions in which *Escherichia coli* was grown. These conditions are modeled as a multiplex network. Specifically, we focus on the transcriptomic layer (microarray data) and on the fluxomic layer (reaction rates at steady state, which we consider as the minimum proxy for the phenotype). The idea is that analyzing growth conditions by focusing only on a single omic level will likely miss complementary information and therefore lead to incorrect evaluations. For instance, considering only gene expression profiles as a response to conditions will miss the actual metabolic response of the bacterium, and will not provide insights into the resulting cellular behavior.

Two main methods have been recently proposed to address the task of integrating multiple data types in an aggregate layer of a multiplex network. iCluster, developed by Shen et al. [6], proposes a joint latent variable model for integrative clustering of multiple genomic datasets. More specifically, tumor subtypes are modeled as unobserved latent variables, which are then estimated from the multiple data types available. Since iCluster does not scale to the entire set of available measurements and therefore needs gene preselection steps, an alternative approach was developed by Wang et al. [7], which is instead based on networks of samples as a starting point for the creation of an aggregate layer. Both methods have been only proposed on genes and, since a genotype-phenotype link is missing, they are not able to target and classify growth conditions.

Unlike the existing methods for network aggregation, we propose a weighted network fusion that takes into account the importance of each layer. This allows us to apply our method to multi-omic genome-scale models where nodes represent environmental conditions and layers represent transcriptomic and phenotypic information. The weight measures the relative importance of each layer, which is quantified by the reliability of the genome-scale model in predicting correct flux rates starting from gene expression data. As a result, our multiplex network approach is able to integrate and elucidate condition similarity in multi-omic genome-scale models, and to be calibrated to the quality of different data types available for a given organism. To test our method, we build a genome-scale model of *Escherichia coli*, starting from the iJO1366 reconstruction [8] and including recently discovered gene-protein associations and a comprehensive set of underground metabolic reactions.

**Fig. 1:**
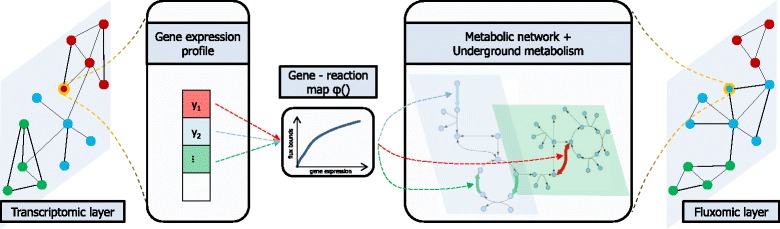
The transcriptomic and fluxomic layers of environmental conditions constitute our multiplex (duplex) network, where nodes are environmental conditions. The real-valued gene-reaction map *φ* converts gene set expression values into flux bounds for the trilevel FBA model of *E. coli* (see *Methods*). For each condition, the gene expression profile is mapped to the metabolic model, and a trilevel linear program is solved to calculate the condition-specific distribution of flux rates, therefore linking gene expression to phenotype. A network of conditions is then built independently in both layers. The multiplex network is then fused into a single network through our weighted network fusion approach. Finally, further learning is performed on the combined network to elucidate relations between conditions

The steps of our method are presented in Fig. 1. To our knowledge, this is the first attempt of multiplex analysis on a genome-scale metabolic network from a condition-based perspective. We take into account condition-dependent omic data and, for the first time, we propose a method for weighted network fusion of omic layers in a multiplex.

## Methods

### An improved model of *Escherichia coli* with underground metabolism and new gene-protein-reaction associations

To obtain the model used in this study, we started from the iJO1366 *E. coli* reconstruction [8]. We then added the full set of underground reactions reported by Notebaart et al. [9]. These underground reactions occur at low rates compared to the rest of the metabolic network, and new metabolites are usually detected with low abundances. Interestingly, the effect of the underground reactions is promptly detected in the newly built genome-scale model, since many underground reactions share metabolites with existing reactions or constitute new pathways leading to precursors for biomass production.

Finally, we included new gene-protein-reaction (GPR) associations uncovered by enzyme promiscuity analyses and failed predictions of gene essentiality [10] (see Table 1). These cases are derived from a workflow applied to aspC, argD and gltA, three genes that were incorrectly predicted as essential genes in the previous genome-scale reconstructions, therefore suggesting missing isozymes responsible for the corresponding reactions. Potential missing isozymes were identified by running BLASTp [11] on the protein sequences, and predictions were confirmed by single- and double-knockout analysis.

**Table 1:** New gene-protein-reaction (GPR) associations added in the *E. coli* model augmented with underground metabolic reactions, according to the experimental studies in [10]

Reaction name	GPR in iJO1366	GPR in this study
Aspartate transaminase	aspC	aspC OR tyrB
Tyrosine transaminase	aspC OR tyrB	aspC OR tyrB OR ilvE
Acetylornithine transaminase	argD OR astC	argD OR astC OR gabT OR puuE
Succinyldiaminopimelate transaminase	argD	argD OR astC OR gabT OR puuE
Citrate synthase	gltA	gltA OR prpC

We remark that adding the underground metabolic reactions and the new GPR associations did not generate detrimental intermediates or reduced flux in those pathways supporting generation of biomass. The inclusion of underground metabolic reactions also increased flexibility and fitness under various growth environments. The resulting model, provided as Additional file 1, contains 1380 genes, 3027 reactions (including exchange reactions), and 2151 metabolites.

### Steady-state analysis of genome-scale models

Various techniques for modelling and simulating metabolic networks have been developed in recent years to better understand bacterial metabolism, as well as to support rational design for reprogramming microorganisms and overproducing biochemical compounds [4]. Arguably, the most successful technique for predicting flux distributions at steady state is flux-balance analysis (FBA) [12]. FBA is based on two main assumptions: (i) homeostatic assumption, i.e. the organism has reached a steady state where the metabolite concentrations are constant and a set of nutrients are being constantly converted to generate biomass; (ii) (multi-level) optimality, i.e. in each state the organism tends to maximize one or multiple objectives, usually related to growth, excretion of biotechnologically-relevant metabolites and important energy-carrying molecules. FBA is suitable for analyzing the flow of metabolites through a metabolic network (e.g. their formation and degradation, transport and cellular utilization).

Let the metabolic network be composed of *m* metabolites with concentration *x*_*i*_, *i*=1,…,*m* and *n* reactions with flux rates *v*_*j*_, *j*=1,…,*n*. The derivative of the concentration of each metabolite can be computed through a linear combination of the input and output reaction fluxes, which produce and consume (respectively) that metabolite. The balance that metabolite concentrations *x*_*i*_ must satisfy is $\frac {dx_{i}}{dt} = \sum _{j=1}^{n} A_{\textit {ij}}v_{j}, \quad i=1,\ldots,m$, where *A*_*ij*_ is the stoichiometric coefficient of the *i*th metabolite in the *j*th reaction. Under steady state conditions $\frac {dx_{i}}{dt} = 0, \ \forall i$, the balance for the i*th* metabolite is $\sum _{j=1}^{n} A_{\textit {ij}}v_{j} = 0$ (homeostatic assumption). Therefore, at steady state, the balance equation is *Av*=0, where *A* is the stoichiometric matrix (*m* rows and *n* columns), and *v* is the vector of the flux rates (metabolic and transport fluxes).

### Quantitative gene expression levels in genome-scale models

Recently, FBA has been integrated with regulatory constraints taken from gene expression data. For a comprehensive evaluation of these methods, the reader is referred to the paper by Machado and Herrgård [13]. The most widely used approach for linking gene expression level and FBA models consists of removing those reactions in the model that are linked to genes whose expression is below a specific threshold. This approach allows a fast implementation of the effect of gene expression variations on the model, but does not provide the necessary level of information needed for a comprehensive and predictive steady-state biological model. Furthermore, it forces the gene expression levels to be mapped onto a binary domain {0,1}, depending on whether the corresponding reaction is active or switched off. Finally, the introduction of the on/off threshold restricts the possible optimal configurations to a few points in a discrete variable space [14]. A further limitation of these methods is that they consider only one objective, or a linear combination of objectives (usually encoded in the biomass reaction); this does not allow one to fully explore the metabolic potential of the organism, where competing goals may lead to a maximized production of a given chemical while simultaneously ensuring high growth rate.

We overcome these limitations by using multi-level linear programming and by modeling the upper and lower bound of each reaction as a continuous logarithmic function of the related transcriptomic data, using METRADE [15]. In this way, the gene expression values are mapped onto the set of all real numbers, and are scaled to become RNA abundances, thus controlling variations of the reaction fluxes. Since the expression data is related to genes while the reactions in the FBA model depend on gene sets, we also provide a function acting as an interface between the data and the model (i.e., between genes and gene sets). Each reaction in a FBA model depends on a single gene set that controls the reaction through AND/OR operators between genes. We formalize these relations using the *min* and *max* operators, which make it possible to convert the gene expression levels *θ* into a *“gene set expression level”θ* [16]. Specifically, we use the following rules valid for the three basic cases of gene set:

(1) $${} \begin{aligned} \Theta(g) &= \theta(g) \qquad\quad\qquad\quad\qquad\text{for single genes}, \\ \Theta(g_{1} \wedge g_{2}) &= \min\{ \theta(g_{1}), \theta(g_{2})\} \quad\text{for enzymatic complexes}, \\ \Theta(g_{1} \vee g_{2}) &= \max\{ \theta(g_{1}), \theta(g_{2})\} \quad\qquad\text{for isozymes}. \end{aligned}  $$

Note that the expression level of nested gene sets is computed by applying these rules recursively.

### Multi-level condition-specific metabolic networks

In the present study, each condition and the corresponding gene expression profile are used as indicators for the activity of the associated reactions in the model, and are therefore mapped onto the model and finally associated with a point in a multi-dimensional phenotypic space. We assume that the genetic level is slower than the metabolic one; the time that chemical reactions within the cell need in order to reach a steady state is usually less than a minute [17], and therefore the steady state is reached faster than the variation of enzyme concentrations caused by changes in the gene expression profile. Furthermore, we account for the fact that the importance of a gene for the metabolism is negatively correlated with the variance of that gene across the experimental conditions [18]. Essential genes are in fact more tightly regulated and evolve slowly, as a high variance of gene expression level of essential genes would affect all the downstream genes and is more likely to be less tolerated in a large interaction network [19, 20]. For instance, essential genes in the metabolic network are those coding for an enzyme controlling a reaction which is upstream of many others (e.g. upstream of the TCA cycle). To this end, we take into account the inverse of the variance of a gene across conditions as a multiplicative factor for the lower and upper bound of the flux regulated by the gene.

Formally, we define the trilevel linear program

(2) $${} \begin{aligned} & {\max} & & h^{\intercal} v \\ & {\text{such that}} & & \max g^{\intercal} v \\ & & & {\text{such that}} & & \max \left\{\,f^{\intercal} v \left| Av = 0, \quad \right.\right.\\ &&&&&\left.\left.V_{i}^{\min} \varphi(\Theta_{i}) \leq v_{i} \leq V_{i}^{\max} \varphi(\Theta_{i}) \right. \right\}, \end{aligned}   $$

where *i* ranges over the reactions (*i*=1,…,*n*), while *f*, *g*, *h* are *n*-dimensional Boolean arrays of weights associated with the three flux rates (entries of the flux array *v*) selected as objectives in the model. *V*^min^ and *V*^max^ are the arrays of default lower and upper-bounds for the flux rates in the model. If *Θ*_*i*_ is the gene set expression of the *i*th gene set (which is associated with the *i*th reaction of the model), the function

(3) $$ \varphi(\Theta_{i}) = \left[1+\frac{\gamma}{{\sigma_{i}^{2}}} \left|log(\Theta_{i})\right|\right]^{\text{sgn}(\Theta_{i}-1)}, \quad i=1,\ldots,n   $$

(and *φ*(*Θ*_*i*_)=1 if *Θ*_*i*_=1) maps the gene set expression value onto the metabolic model. The sign operator is defined as sgn(*Θ*_*i*_−1)=(*Θ*_*i*_−1)/|*Θ*_*i*_−1|. ${\sigma _{i}^{2}}$ is the variance of the *i*th gene set, computed from the variance of the genes involved using the rules (1) defined to map the gene expressions to the gene set expressions. *γ* is the weight for the variance, and constitutes the reliability of the variance as an indicator of the importance of the genes in the model.

The reasons for choosing this mathematical structure are as follows. Logarithmic and multi-layer processes are not uncommon in biology [21, 22], where many processes display a multiple layer architecture in order to produce amplification in cascades (e.g. blood clotting [23] and MAP kinase cascades [24]). Although the correlation between gene expression and metabolic phenotype is still a matter of debate, our assumption of a logarithmic map *φ* is supported by recent evidence that, although protein synthesis rate increases with increasing mRNA abundance, the rate of increase is lower for high mRNA abundance [25]. Furthermore, *φ* provides full compatibility with the above-mentioned Boolean on/off approaches, as it approaches zero when the expression level approaches zero. Being an approximation of a linear function around 1 (which in our method represents the wild-type gene expression level for *E. coli*), we also capture the property of roughly linear relation between gene expression and enzyme activity for the wild-type *E. coli* [26, 27].

The definition of *φ* is also useful when searching for optimal gene expression values in a given condition [28]. Such algorithm would not be encouraged to search for unrealistically high values of gene expression levels, since this would not be converted into weak constraints (as it would happen with, e.g., a linear map). As introduced above, the parameter *γ* is quantifying the role attributed to the variance as an indicator of the importance of a gene (we assume that low variance across different conditions means high importance). By increasing the parameter *γ*, we increase the ability of the gene expression values to fine tune the final reaction rates. The method is robust with respect to perturbations of *γ*, while variations of orders of magnitude lead to an increase of the metabolic sensitivity to the different environmental conditions.

Here we use the logarithmic map (3) to set constraints for the metabolic model, then we solve the linear program (2) to find the flux distribution. While suggesting a logarithmic map, we remark that *φ* is fully adjustable depending on the type of model and on further data available on protein abundance, which would allow a reaction-specific definition of *φ*. Given existing evidence for a logarithm-like regulation of reaction rates, when incorporating additional experimental data into the model, we suggest keeping the logarithmic map and calibrating the base of the logarithm and *γ* as adjustable parameters. With good accuracy, our method enables the prediction of the condition-specific growth rate measured experimentally. It also outperforms existing methods based on linear associations between gene expression and reaction flux bounds. Overall, it shows high correlation between experimental and predicted rates (see *Results*).

### Multiplex networks to model omics response to environmental conditions

Multilayer networks are particular types of networks used to model interactions between components in a system where different channels of connectivity are explicitly taken into account. A channel is associated with a layer, and each node may have different interactions with the other nodes, depending on the layer where these interactions take place [29, 30]. Our focus is on investigating similarity between environmental growth conditions by analyzing the *E. coli* response from a multi-omic perspective. To this end, we link the two layers through a gene-expression driven trilevel linear program. This multi-level structure accounts for the fact that the actual bacterial response to any environmental condition is highly dependent on multiple cellular objectives that the bacterium is required to meet [31, 32].

Since we are interested in analyzing conditions and finding condition similarity from the transcriptomic and fluxomic viewpoints, we construct a multiplex with these two layers, where nodes are environmental conditions. The edges between nodes in each layer represent a measure of similarity between conditions in terms of gene expression profile and metabolic flux profile respectively. In order to obtain a global picture of similarity between conditions, we aggregate both layers in a single-layer network through a weighted network fusion approach. After aggregating the system into a single network, we adapt measures on graphs with the aim of identifying clusters of similar conditions, where the similarity takes into account the two omics (transcriptomic and fluxomic) and the metabolic network.

### Similarity network fusion (SNF)

#### Motivation

In recent years, complex networks have come to increased prominence as a dataset type. Many types of information, particularly in biology, are best represented in terms of pairwise interactions (edges) between entities (nodes), which can be a particularly compact form for large systems where only a small proportion of elements have interesting interactions.

As more datasets are gathered and stored as complex networks, it becomes increasingly common to find multiple network datasets (edge sets) that refer to the same entities (node sets). We might suppose that sometimes these network datasets are different reflections of some common underlying network, and we may wish to integrate the known dataset to discover the structure of the underlying, unknown, dataset. This is the purpose of Similarity Network Fusion (SNF) [7].

#### Definition of *W*

*W* are the base similarity networks that need to be integrated. In each layer, if the entries of *W* (edge weights) are already representing similarity between nodes of the network, then they only need to be normalized to be positive and in [ 0,1], e.g. by squaring and dividing by the maximum. For instance, social networks are often natively similarity networks, where edge weights are counts of various friendly events between individuals.

However, distance is probably more common than similarity as an edge weight type. When we have distances, we must transform them to similarities, with Wang et al. [7] suggesting a scaled exponential similarity kernel:

(4) $$ \mathbf{W}(i,j) = \text{exp}\left(- \frac{\rho^{2}(x_{i},x_{j})}{\mu \epsilon_{i,j}}\right).  $$

#### Definition of *P*_0_

*P*_0_ are the starting state of the networks, which will be modified as the algorithm iterates. As such, they start as normalized versions of *W*. Appropriate normalizations are:

(5) $$ \mathbf{P_{0}} = \mathbf{D}^{-1}\mathbf{W}  $$

where

(6) $$ \mathbf{D}(i,i) = \sum_{j} \mathbf{W}(i,j),  $$

or

(7) $$ \mathbf{P_{0}}(i,j) = \left\{ \begin{array}{ll} \frac{\mathbf{W}(i,j)}{2 \sum_{k \neq i} \mathbf{W}(i,k)} & \text{if}\; j \neq i \\ \frac{1}{2} & \text{if}\; j=i \end{array}\right.  $$

which is more robust to numerical instabilities.

#### Definition of *S*

*S* are local similarity matrices for the networks. These are modified similarity matrices such that only the *K* nearest neighbors of a node have nonzero similarity. If *N*_*i*_ is the set of neighboring nodes of *x*_*i*_ (including *x*_*i*_), then we define local similarity by:

(8) $$ \mathbf{S}(i,j) = \left\{ \begin{array}{ll} \mathbf{W}(i,j) & \text{if}\; j \in N_{i} \\ 0 & \text{otherwise} \end{array}\right.  $$

#### Core operation

The core network integration procedure consists of iteration of the following update equation:

(9) $$ \mathbf{P}^{(v)}_{n+1} = \mathbf{S}^{(v)}_{n} \times \left(\frac{\sum_{k \neq v} \mathbf{P}^{(k)}_{n}}{m-1} \right) \times \left(\mathbf{S}^{(v)}_{n}\right)^{T}\!, \quad v=1,\ldots,m,   $$

where *v* is the index of each of the *m* layers, *k* ranges over all layers except for the one under consideration, *n* is the iteration number, $\mathbf {P}^{(v)}_{n}$ is the similarity matrix, and $\mathbf {S}^{(v)}_{n}$ is the local similarity matrix, both referred to the *v*th layer at the *n*th iteration.

For a set of networks (layers of a multivariate network), this iteration repeatedly makes each network more similar to the others until they converge to a single integrated network.

#### Code listings

For those readers who find source code more illuminating than equations, the file https://github.com/maxconway/SNFtool/blob/master/R/SNF.R is a good place to start.

### Biased similarity network fusion

**Fig. 2:**
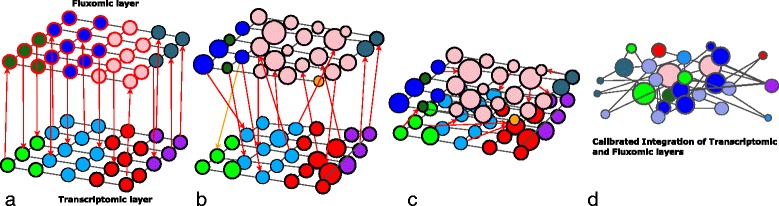
Visual schema of the multiplex fusion algorithm. The bottom layer in panels (**a**-**c**) represents the transcriptomic information, while the top layer represent the fluxomic information. Each circle represents a feature (parameter) of the system, which we consider as an environmental condition. Black connectors represent parameter relationships; red links represent the mapping from gene expression to phenotype through the metabolic map *φ*, and also convey the information related to the message passing method for the SNF approach. The four panels represent: **a** ideal scenario; **b** more likely real scenario; **c** fusion proximity; **d** fusion and reduction of parameter complexity, performed through measures on single-layer networks (e.g. clustering or community detection)

To combine the multiple gene expression and phenotype networks together we propose a technique based on SNF [7]. SNF takes a number of networks with the same set of nodes (i.e., layers of a multiplex network), and iteratively alters each network to resemble the others, until all the networks converge to an aggregate network (Fig. 2). The key idea in this study is to aggregate weighted layers of environmental conditions, measured in the transcriptomic and fluxomic levels, each constituting a layer of the multiplex. To this end, we develop a weighted similarity fusion approach that allows us to account for the quality of the metabolic reconstruction when linking gene expression and phenotype. We therefore allow the use of a bias between layers to reflect this. For instance, in the network fusion process, if the predictive capability of the genome-scale model is high, one is able assign more importance to the phenotypic data rather than to the transcriptomic data.

Since our multi-omic model and trilevel formulation provide phenotypic flux rates from gene expression profiles associated with growth conditions, one may object that the transcriptomic layer should carry no weight in the fusion process. However, we remark that the fluxomic layer is a result of predictions of the metabolic model, and therefore it should not be considered as the only indicator of the response to a given condition. In fact, in applications to genome-scale models (as in this study), we suggest setting the weight as the level of confidence in the model itself.

In order to perform network fusion, we create a similarity matrix of environmental conditions in two different layers: transcriptomics and fluxomics. First, we remove gene expressions and fluxes for which less than 10 % of values were known and finite. Then, we divide each expression and flux into 20 quantiles, and replace each value by their quantile number, to deal with the high kurtosis; finally, we calculate the Euclidian distances between nodes (conditions), and we find similarities **P**_*ij*_ in each layer *v* by exponential negative squared Euclidean distance

(10) $$  \mathbf{P}_{ij}^{(v)} = e^{-\left[d_{ij}^{(v)}\right]^{2}},  $$

where $d_{\textit {ij}}^{(v)}$ denotes the Euclidean distance between the two arrays representing conditions *i* and *j* in the *v*th layer.

Let **P** be the multiplex network of similarity between environmental conditions. **P** is computed, using (10) on *m*=2 omic levels, i.e., from the distance between gene expression arrays in the transcriptomic layer, and from the distance between flux rate arrays in the phenotype layer. The central equation of standard SNF is Eq. 9, which is iterated to actually conduct the fusion process, namely a finite number of message passing steps in which the *m* layers co-evolve. Eq. 9 describes the update step for each of the *m* status matrices **P**^(*v*)^, representing similarities of conditions in each layer. These matrices are initialized as a normalized form of the similarity matrices of the *m* networks. **S**^(*v*)^ are kernel matrices, giving a normalized form of similarity only to the *K* nearest neighbors.

Note that Eq. 9 computes an unweighted mean over **P**^(*k*≠*v*)^. To introduce a weighted network fusion, we introduce a vector of biases, $\mathbf {b} \in \mathbb {R}^{m}$, which are used to alter the update step and take a larger input from some of the *m* layers than others. Then, we replace Eq. 9 by performing the following update step for each layer:

(11) $${} \mathbf{P}^{(v)} = \mathbf{S}^{(v)} \times \left(\frac{\sum_{k \neq v} \left(\mathbf{P}^{(k)} \times \mathbf{b}_{k} \right)}{(m-1) \times \sum_{k \neq v} \mathbf{b}_{k}} \right) \times \left(\mathbf{S}^{(v)}\right)^{T}, \,\,v=1,\ldots,m.   $$

In addition to introducing a layer bias, we added convergence detection based on the first and second discrete derivatives, and parallelized each iteration for an *m*−fold performance increase when fusing *m* networks. Our weighted network fusion method is freely available at https://github.com/maxconway/SNFtool.

## Results

### Estimating optimal bacterial response in multidimensional phenotypic spaces

A common assumption in systems biology is that microorganisms tend to shape their metabolic network in order to maximize the growth rate (biomass). However, whether the biomass is the right objective for the analysis of the metabolism is still a matter of debate [33]. Furthermore, there is increasing evidence that bacteria have to cope with multiple, sometimes competing, objectives that must be fulfilled simultaneously [34]. It is also likely that evolution has shaped cells in order to reach an optimal trade-off between all their objectives [35]. This suggests that a single-objective approach, the maximization of the growth rate, may not be appropriate in many systems biology applications.

To enable a multi-objective view of the metabolism, we base our method on a multi-level linear program aimed towards classifying the bacterial response associated with external or growth media conditions in any multidimensional objective space chosen by the researcher. Our pipeline can be used to design environmental conditions that likely lead to a desired phenotype. Due to its real-valued kernel, it is well suited for applications involving various perturbations, such as different codon usage bias or continuous genetic manipulations.

**Fig. 3:**
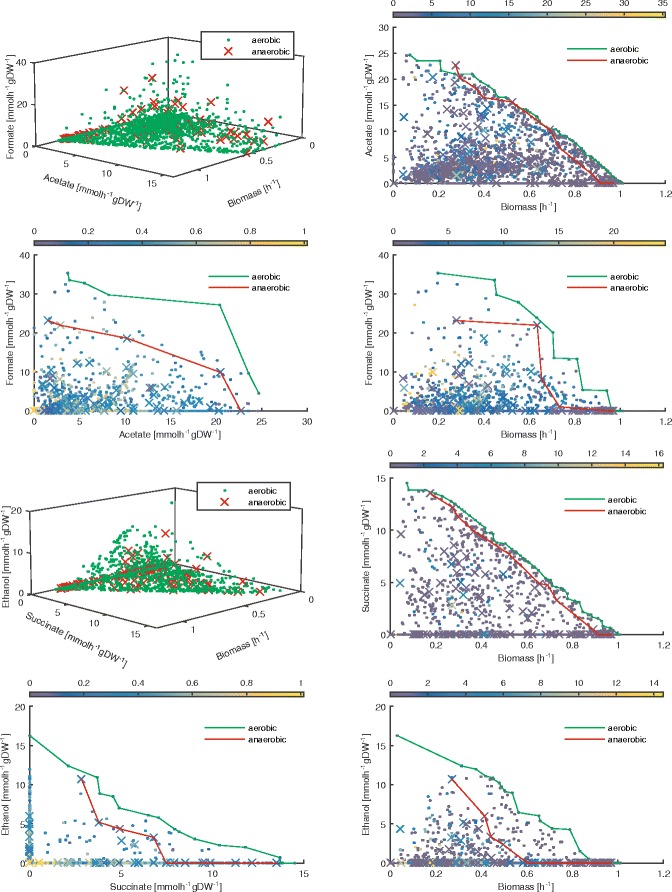
The 2369 Colombos gene expression microarray profiles mapped to the tridimensional space of objective functions biomass-acetate-formate (top four panels) and biomass-succinate-ethanol (bottom four panels) using trilevel linear programming (Eqs. (2-3)). Each gene expression profile is translated into flux bounds using (3); then, the trilevel problem (2) is solved with biomass-acetate-formate and biomass-succinate-ethanol as objectives, thus obtaining a point in each of the two objective spaces. In both objective spaces, we show the conditions mapped to the full space (*top left*), and the projections to the three two-dimensional subspaces: first-second objectives (*top right*), second-third objectives (*bottom left*), first-third objectives (*bottom right*). We also find the trade-off between the two objectives shown in each subspace, across the sets of aerobic and anaerobic conditions. The color scale shows the value of the third objective in each point. Among the 2369 conditions (obtained with different pH, antibiotics, heat shock, glucose concentrations), 128 conditions are anaerobic. The plot also shows the subspace where *E. coli* operates in both the objective spaces selected and allows cross comparing the metabolic flexibility when production of different metabolites is required simultaneously

We apply our multi-level linear program (see *Methods*) by mapping the Colombos v2.0 compendium of expression data [36], which includes *E. coli* microarray profiles for 2369 measured conditions, to an *E. coli* model that includes underground metabolism (provided as Additional file 1). The expression profiles have been measured using microarrays (Affymetrix *E. coli* Genome 2.0) with raw hybridization of intensities, as well as RNA-seq (Illumina MiSeq) with short read sequences, therefore obtaining homogenized expression profiles. Conditions have been fully annotated through manual curation. In Fig. 3, we show the 2369 conditions projected to the three-dimensional spaces biomass-acetate-formate and biomass-succinate-ethanol using Eqs. (2–3) and choosing the triplet of objectives using *f*,*g*,*h* in Eq. (2). Each space is then further projected to the three two-dimensional subspaces. As a result, we map the set of experimental conditions to a set of metabolic networks, each of which yields a specific amount of output metabolites. The red and green lines indicate the trade-off between the two objectives shown, across the sets of aerobic and anaerobic conditions. The full list of Colombos conditions and the corresponding flux rates predicted using trilevel linear programming are reported as Additional file 2.

When maximizing for acetate and formate production, *E. coli* shows greater variability and different outcomes in different conditions. Conversely, when maximizing for succinate and ethanol production, less conditions are able to ensure high succinate or ethanol production. Interestingly, many conditions able to ensure high ethanol production are anaerobic. In the acetate-biomass space, the two trade-off curves intersect, due to the fact that in one anaerobic condition *E. coli* is able to produce 22.72 mmol h ^−1^ gDW ^−1^ of acetate while keeping a growth rate of 0.27 h ^−1^. This configuration is not reached in aerobic conditions. We also note that the presence of oxygen strongly affects the bacterial response in the ethanol-biomass space (e.g. compared with the formate-biomass, acetate-biomass and succinate-biomass spaces).

The distinction between aerobic and anaerobic conditions is of great metabolic importance, and it is reliably recorded in the Colombos condition database. From Fig. 3, we note that under almost all circumstances, the aerobic Pareto front covers more “metabolic space”, since adding oxygen allows more possible metabolic configurations. Furthermore, a large number of conditions excrete none of each of the byproducts. This is also unsurprising, since excreting these byproducts effectively means excreting energy, which has obviously been selected against. We also note that, in the graphs showing biomass and byproduct excretion, for all byproducts except for succinate, there is a clear scarcity of conditions in the high byproduct, low biomass region (top left). We hypothesize that this is due to the fact that if the overall metabolism of the cell is slow, i.e. low biomass, it will be more difficult for the cell to spare resources to give high byproduct production while still remaining alive.

Interestingly, only a few conditions are able to produce simultaneously succinate and ethanol. In fact, the succinate-ethanol and biomass-ethanol subspaces are where the difference between aerobic and anaerobic condition is more evident. This is mainly due to the difficulty in producing ethanol and high amount of biomass in anaerobic condition (maximum 10.71 mmol h ^−1^ gDW ^−1^ of ethanol, with a biomass of 0.27 h ^−1^). Indeed, the trade-off in anaerobic condition is far from the trade off in aerobic condition especially in the low-biomass and high-biomass intervals. This is not observed in the succinate-biomass phenotypic space, where the aerobic trade-off is close to the aerobic trade-off even at high biomass production rates.

### Validation on a phenomics dataset of growth conditions

**Fig. 4:**
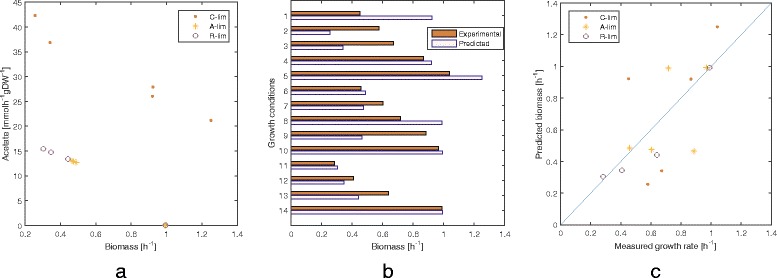
Validation on the phenomics dataset of growth conditions by Hui et al. [37]. The dataset includes five *C-lim* conditions (titrated catabolic flux through controlled inducible expression of the lacY gene), five *A-lim* conditions (titrated anabolic flux through controlled expression of GOGAT), and four *R-lim* conditions (inhibition of protein synthesis with chloramphenicol, an antibiotic). **a** The 14 gene expression profiles are mapped to the biomass-acetate space of flux rates. Each gene expression profile yields a condition-specific metabolic network, solved as a bilevel linear program with biomass-acetate as objectives, thus obtaining a point in the objective space. The *C-lim* experimental conditions allow for more acetate production while ensuring higher growth rate and greater variability in different conditions. **b** Measured growth rates are compared with those predicted by our method in the 14 growth conditions. **c** We obtain a good overall correlation between our predicted values and the measured growth rate, with Spearman’s *ρ*=0.678 (*p*-value=0.008) and Pearson’s *r*=0.680 (*p*-value=0.007). The diagonal “predicted = experimental”, representing the ideal outcome, is also shown for comparison

In order to validate our method for associating a predicted growth rate to each condition, we consider a recent phenomics dataset of 14 growth conditions [37]. On the new model of *E. coli* we obtain encouraging results in predicting the growth rate from a set of expression profiles associated with different experimental conditions (Fig. 4a). Specifically, we obtain a strong correlation between predicted and measured growth rates (Pearson’s *r*=0.680, *p*-value=0.007, and Spearman’s *ρ*=0.678, *p*-value=0.008). A comparison between predicted and measured growth rates is reported in Fig. 4b. As shown in Fig. 4c, the best results are obtained with the subset of conditions representing inhibited protein synthesis by supplying chloramphenicol to the growth medium (*R-lim*).

We report that our method consistently outperforms the methods for integration of gene expression data in FBA that employ a linear map between gene expression values and the multiplicative factors for the flux bounds (an approach used, e.g., in [38]). For instance, with the dataset used in this study, linearly increasing and decreasing bounds according to gene expression data only leads to a Pearson correlation of 0.13 (*p*-value=0.67) and, surprisingly, to a negative Spearman correlation of −0.19 (*p*-value=0.52) between predicted and measured values.

The inclusion of the underground reactions causes a relevant effect in the prediction of growth and production of additional objectives performed through flux-balance analysis. This is due to the fact that underground reactions share several metabolites with the iJO1366 reactions. Some underground reactions also take part in pathways leading to biomass precursors.

### Multi-omic and multi-condition network fusion

In the transcriptomic layer, a condition is represented by a gene expression array; conversely, in the phenotypic layer, it is represented by a flux rate array. Our phenotypic flux data and, to a lesser extent, Colombos expression data displayed high kurtosis, indicating that both layers display many outliers. We do not remove outliers for two main reasons: first, in some cases this would remove most of the data; second, this represents a biologically reasonable all-or-nothing regulation, as one would see in a bistable system created by positive feedback in regulation (for instance [39]).

**Fig. 5:**
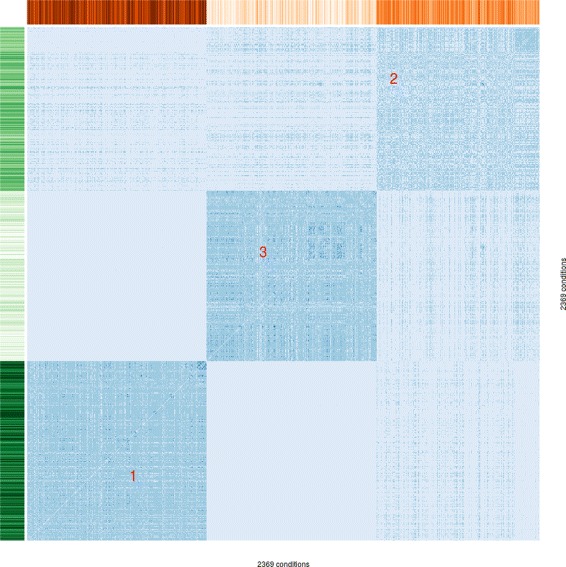
Heat map of the similarity matrix of the fused network from our case study, arranged by spectral clustering into three components. The *x* and *y* axes represent the 2369 conditions, while the intensity of the colors in the center represent the similarity between each of the pairs of x and y conditions. The red numbers are cluster labels, from 1 (highest flux rates) to 3 (lowest flux rates). The intensity of the orange and green bars on the top and side represent 5-deoxyribose exchange rate and biomass production, respectively. The rate of both these fluxes can be partitioned and can be used with high confidence to provide clear distinctions between the clusters of conditions. The partitioning process we used was able to provide a similarly clear distinction in both dimensions using each of the fluxes reported in Table 2

After constructing the multi-omic similarity network (see Methods), we fuse together the phenotype and gene expression layers with a *K* value of 500 (number of nearest neighbors) and a phenotype-transcriptome bias of 2:1. We then assess the resulting network by conducting spectral clustering and plotting a heat map. We find that three clusters best represent the data. While moving to larger numbers of clusters could decompose the center cluster slightly, it sacrifices most of the contrast at the cluster boundaries. Figure 5 shows the heat map resulting from the spectral clustering performed on the fused network, whose outcome is reported in [40].

**Table 2:** Reactions identified as important classifiers by the decision-tree-based prediction of clusters

Reaction name	Importance (rounded %)	Cluster 1 LB	Cluster 3 UB
Biomass generation	17	1.009824	1.0079
CHEBI:44800 production	17	0.001353165	0.001350586
5-deoxyribose production	17	0.0002332694	0.000232825
p-Cresol production	17	0.0002251908	0.0002247617
CHEBI:16490 production	17	2.019649e-06	2.0158e-06
Other	15		

To detect those fluxes and expressions that are most indicative of the three configurations, we employed a regression technique based on recursive partitioning [41]. This suggested a number of decision trees with only two rules splitting on flux values, each able to assign the correct cluster in 97 % of cases. We validated these fluxes as genuinely important via bootstrapping: on repeated samples of 80 % of the data, the same fluxes were detected as important. The fluxes we found to be closely associated with clustering are reported in Table 2. Of these, we can immediately associate biomass generation and 5-deoxyribose production with growth rate. The other exchange reactions may have specific relationships with growth rate configurations, or their detection may be due to a general correlation between rates of bacterial growth and excretion of byproducts. The orange and green bars in Fig. 5 show how effective these fluxes are in partitioning the data into clusters. Interestingly, two out of five predictors are underground reactions.

## Discussion

Multi-layer networks are increasingly being associated with microorganisms, with a rapidly growing number of applications in systems biology [42]. Bacterial metabolism can itself be thought of as a multi-layer network. This is due to the fact that a microorganism builds complexity in time and space by evolutionary increasing its capability of performing computation through chemical reactions over time, increasing the so called “bacterial computational capability” [43], usually through late transfer of modules that have spread across bacterial species. Bacteria have therefore hierarchical regulation machineries for processing gene expression to RNA, RNA to proteins, proteins to post-translational modified (or complexes of) proteins, and finally complexes of elements to structural organized macrostructures.

While single-layer networks are able to model interactions between components, their strongest assumption, i.e. all the interactions between two nodes can be mapped as a single link, does not always hold in biology. Interactions between the same nodes can occur at different levels and in different settings [44, 45]. For instance, in case of a compendium of environmental growth conditions for a microorganism, the bacterial response is measured at different omic levels. In this study, we analyzed the transcriptomic and fluxomic levels.

## Conclusion

When we are interested in classifying similarity among growth conditions, and in finding communities structure within their network, we must account for the different omic levels where the response to these growth conditions can be measured.

After building a genome-scale reconstruction of *Escherichia coli* that takes into account underground metabolism, we map a set of growth conditions to the biomass-acetate-formate and biomass-succinate-ethanol spaces of flux rates. This allows us to estimate the metabolic potential of *E. coli*, and to predict regions of the fluxomic space where the bacterium operates in different conditions. As a result, each condition is represented by a gene expression profile in the transcriptomic layer, and by a flux profile in the fluxomic layer. In both layers, a network of conditions is built taking into account similarity between gene expression profiles and predicted flux rates respectively. We then fuse the resulting multiplex network into a single-layer network, by introducing a bias to represent the weight put on the phenotype layer rather than on the gene expression layer. This weight should be related to the accuracy of the genome-scale metabolic reconstruction. For instance, for large and exhaustive models (e.g. the *E. coli* adopted in this study), the information we gather after taking into account the predicted phenotype is likely to be more detailed than merely considering the gene expression levels.

Our multi-omic network fusion method builds on SNF to allow us to: (i) specify a bias between layers; (ii) calculate a similarity matrix in a robust fashion despite extremely high kurtosis distributions; and (iii) attribute the clusters in the fused similarity matrix to a subset of dominant reactions.

Integrating multiplex networks of omic levels in combination with metabolic models serves as an indicator of the behavior of the phenotype across the phase-space of all possible gene expression profiles, and their associated metabolic networks. This paves the way for the high-level topological understanding of multi-omic and multi-condition models. Assigning a specific metabolic model to each experimental condition proves useful in a number of applications. For instance, using the phase-space of conditions, one can predict and investigate metabolic regions in which the bacterium can work under many experimental settings, as well as unapproachable regimes, or regions in which it can grow only in specific conditions. Furthermore, given topological information on the space of experimental conditions, one can infer the position of non-tested or incomplete expression profiles.

Multi-omic data modelling will also provide insights into the complexity of regulatory metabolic mechanisms. However, such complexity could be meaningfully analyzed and used in metabolic engineering only through comparative analysis of the organismal response to thousands of environmental conditions. It is noteworthy that this approach resembles the deep phenotyping techniques used in medicine.

## Availability

The datasets supporting the conclusions of this article are included within the article (and its additional files). Our weighted network fusion method for genome-scale models is freely available at https://github.com/maxconway/SNFtool.

## Additional files

Additional file 1
**The improved model of** ***Escherichia coli*** **with underground metabolism.** The model is provided as a MATLAB file compatible with the COBRA toolbox. It includes the reactions in the latest iJO1366 reconstruction, as well as underground metabolism and new gene-protein-reaction associations. The resulting model contains 1380 genes, 3027 reactions (including exchange reactions), and 2151 metabolites. (MAT 214 kb)

Additional file 2
**Flux rates of Colombos conditions.** Full list of Colombos conditions and the corresponding flux rates predicted using trilevel linear programming. (XLSX 364 kb)

## Abbreviations

SNF: Similarity network fusion
FBA: Flux balance analysis
GPR: Gene-protein-reaction
LB: Lower bound
UB: Upper bound
